# Automatic Region-Based Brain Classification of MRI-T1 Data

**DOI:** 10.1371/journal.pone.0151326

**Published:** 2016-04-20

**Authors:** Sepideh Yazdani, Rubiyah Yusof, Alireza Karimian, Yasue Mitsukira, Amirshahram Hematian

**Affiliations:** 1 Centre for Artificial Intelligence and Robotics, Malaysia-Japan International Institute of Technology (MJIIT), University Technology Malaysia, Kuala Lumpur, Malaysia; 2 Department of Biomedical Engineering, Faculty of Engineering, University of Isfahan, Isfahan, Iran; 3 Department of System Design Engineering, Faculty of Science and Technology, Keio University, Kyoto, Japan; 4 Department of Computer and Information Sciences, Towson University, Towson, Maryland, United States of America; Stanford University Medical Center, UNITED STATES

## Abstract

Image segmentation of medical images is a challenging problem with several still not totally solved issues, such as noise interference and image artifacts. Region-based and histogram-based segmentation methods have been widely used in image segmentation. Problems arise when we use these methods, such as the selection of a suitable threshold value for the histogram-based method and the over-segmentation followed by the time-consuming merge processing in the region-based algorithm. To provide an efficient approach that not only produce better results, but also maintain low computational complexity, a new region dividing based technique is developed for image segmentation, which combines the advantages of both regions-based and histogram-based methods. The proposed method is applied to the challenging applications: Gray matter (GM), White matter (WM) and cerebro-spinal fluid (CSF) segmentation in brain MR Images. The method is evaluated on both simulated and real data, and compared with other segmentation techniques. The obtained results have demonstrated its improved performance and robustness.

## 1 Introduction

Brain segmentation in magnetic resonance image (MRI) plays an important role in quantitative analysis in clinical research, such as surgical planning and drug therapy evaluation, disease detection, and also measurement of brain size, shape and temporal change. In particular robust and accurate method for medical image segmentation is a research topic, which has been one of the problems in medical image analysis. Most procedures rely on manual segmentation method, which is exceedingly time consuming, labor intensive, highly subjective and impractical for large-scale group research. In addition, these techniques suffer from inter- and intra-observer variability. Therefore, automatic segmentation of brain images into three constructing tissues of the brain, white matter (WM), gray matter (GM) and cerebro-spinal fluid (CSF) has become a rapidly growing field of study. Classification of brain image exclusively into distinct tissue types, however, is difficult task due to complex anatomical structure of brain tissues as well as some artifacts such as partial volume effects (PVE), bias field, intrinsic tissue variation and noise [1].

In MRI, bias field is typically caused by non-uniformities in the RF field during acquisition, which is a low frequency smooth undesirable signal [2]. The result is a shading effect where the pixel/pixel intensities of the same tissue type vary over the image and lead to segmentation inaccuracy [1, 3]. Another problem is that due to finite resolution of the imaging devices, a single pixel may contain mixture of tissue types, which is known as PVE. The PVE can be problematic in small structures or highly convoluted regions of the brain. Therefore, it is highly desirable to apply corrected image classification when performing longitudinal studies and group comparison based on a large volume of image data. In the literature there are many procedures aiming to estimate and compensate these kinds of artifacts. There has been a wide range of automatic MRI segmentation techniques proposed in the literature. For example, the thresholding approach that partition the brain image applying deterministic values that divide tissue types according to intensity gray levels. Warfield, et al. have developed image segmentation method, which integrates the techniques of K-nearest neighbors and image registration. The fuzzy-based techniques generalize the K-means approach for soft segmentations such that each pixel can be assigned to more than one type of tissue. The aforementioned techniques all belong to the category of nonparametric segmentation techniques [4–6]. Other branch of segmentation techniques relies on statistical methods. These methods assume that mixed pixel intensities demonstrate different distinct tissue types, and individual pixels are assigned to various groups through modeling the intensity histogram as a mixture of probability distributions. For example the EM algorithm was proposed by Wells et al. that iterates between the estimation of tissue class probability [7, 8]. The main shortcoming of EM based techniques is that they are based on symmetric Gaussian distribution model for the intensity distribution of brain images that is not true in the real MRI [9]. The experimental results demonstrate that the distributions of tissue intensity do not exactly demonstrate a normal Gaussian distribution, particularly for noisy images [10]. Usually in real MR Images, the intensity distributions of brain tissues can vary asymmetrically in these images [4, 9, 11]. Consequently the intensity of individual tissues may display skewed or spread shapes between brain images that may not be well fitted by a Gaussian shape [4, 11, 12]. For example the CSF intensity on T1 brain images usually spread across a wide range at the lower end of the histogram and display an overlap with the GM tissue [4, 9, 11].

Generally, one of the main drawbacks of classifiers is that they do not contain contextual information [13]. One solution is using Markov random fields (MRFs), which is a statistical model in the category of random field methods. Wells et al. [14] firstly introduced MRFs for brain images segmentation using a nonparametric Pazen-windowing model. Zhang et al. have developed a hidden MRF model to achieve a similar purpose [4]. As MRF is a pixel classification technique [3], parameter estimation and optimization are often computationally expensive. Another drawback of MRF is its inability to keep topological knowledge.

Atlas-based segmentation methods are powerful tools for MRI segmentation when a template is available [15, 16]. A brain atlas consists of a typical high-resolution brain structure with coordinate labels, anatomical labels and sometimes functional labels. This atlas is then applied as a reference for analysing new MR images. Atlas-based algorithms consider the segmentation problem as a registration problem [15]. The basic idea of these methods is that a transformation can be found that registers atlas image in which structures of interest have been labeled to the volume to be segmented. If such a transformation can be estimated, tissues labeled in the atlas can simply be projected onto the image of interest. A difficulty with the atlas-based methods is to define an accurate registration, especially for complex structures [15, 17, 18]. Statistical Parametric Mapping method (SPM), is freely available to the brain imaging community for common analysis scheme across laboratories. Brain tissue classification in SPM is an iterative algorithm that requires the brain images to be registered with tissue probability maps. After registration step, these maps define the prior probability of several tissue classes being found at each position in MR image. The performance of SPM package is not influenced by bias field as much as noise, but it is very influenced by noise.

Edge-based methods, which mostly rely on deformable models, use the assumption that pixel values change rapidly at the boundary between two regions. However, the actual region edges should be closed curves. Thus, some post-processing methods, such as smoothing, edge tracking and gap filling, should be conducted to obtain the closed region edges [19].

Region based algorithms use the assumption that adjacent pixels in the same area have similar visual features such as texture, gray level or color value. Region growing is a powerful technique to tackle problematic segmentations that is controlled by a number of initial seeds. The performance of this method depends on the selected homogeneity criterion. Given the seeds, the region growing method tries to find an accurate segmentation of images into different areas with the property that each connected component of a region meets exactly one of the seeds. Furthermore, high-level knowledge of the image components can be extracted from the choice of seeds. Some of the early works on region growing method was described by Cline et al. who used seed growing to extract the surface of the brain. In this method, firstly, a seed point is selected. Then, the neighboring pixels are compared to the first seed, and added to the region if they fulfill some similarity criteria.

The region growing method can work efficiently in medical image segmentation if one can guarantee optimal initial seed point and threshold criterion used to stop growing outside a region. In seeded region growing method (SRG), seed selection is crucial, often done by hand in medical image processing [20, 21]. However, the SRG techniques suffer from the problems of automatic seed generation and pixel sorting orders for labeling [20]. In the literature most of the region growing methods require the seed point be selected manually in advance. In order to make the segmentation process fully automatic, it is necessary to develop an automatic seed point selection approach. We presented an algorithm to automatically select a seed point for the segmentation. Since in the proposed technique, the task of choosing seed points has been automated, the method does not need user interaction to choose the first seed point. Another characteristic of the proposed algorithm is that MR images have been normalized before the segmentation process using a new local normalization method. The balance of the paper is organized as follows. In Section 2, we presented the overview of the proposed method. Experimental results and validation is presented in section 3. Finally, conclusions are drawn in Section 4.

## 2 Methodology

**Fig 1:**
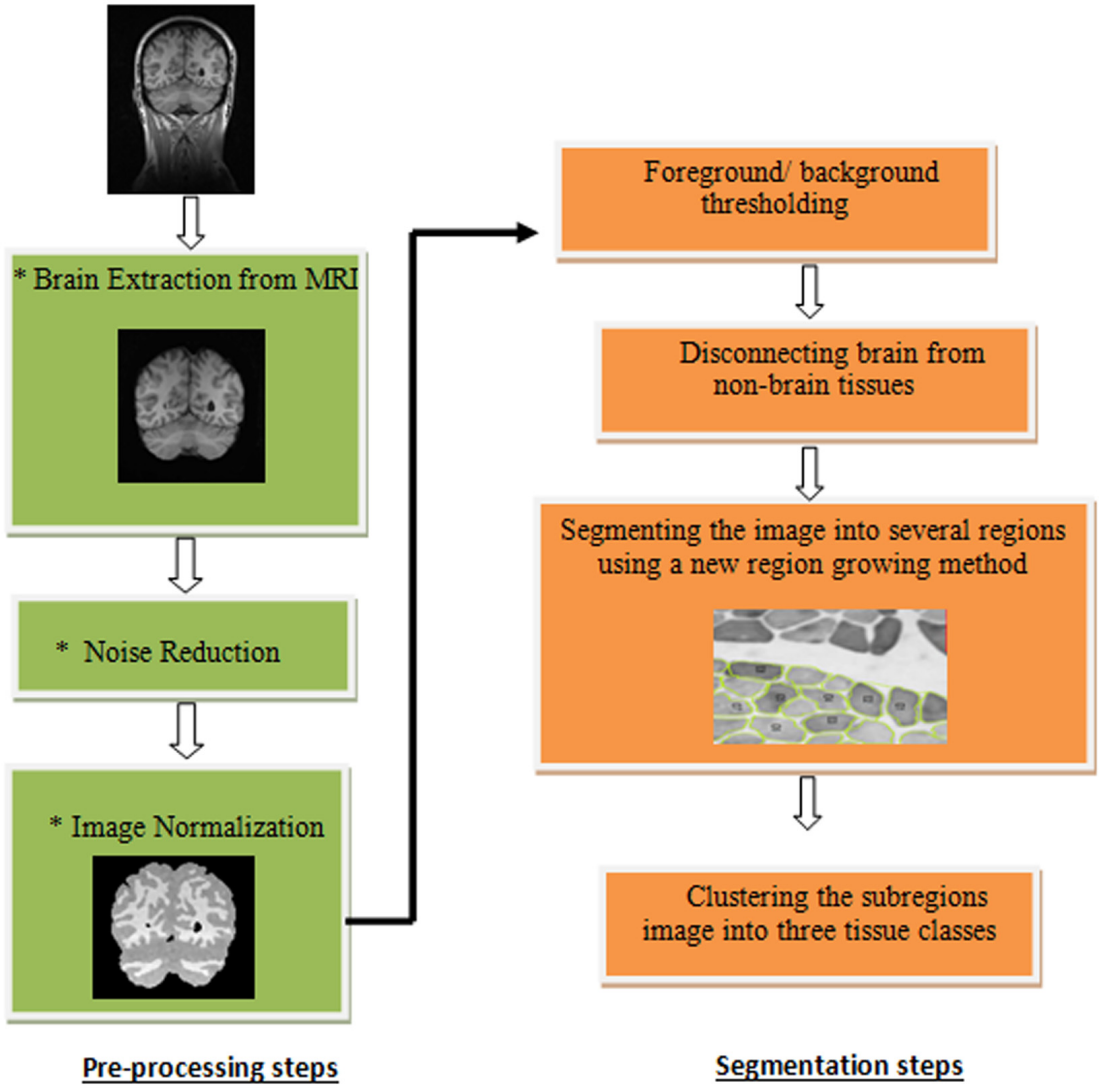
General overview of the region based technique.

In this study, an automatic segmentation method is proposed to segment brain images into main tissue classes. To compensate MRI artifacts the preprocessing steps were applied prior to actual segmentation, which are explained as follows. In the preprocessing step we proposed a new normalization method to compensate bias field effect in MR Images. The proposed segmentation method is a new hybrid algorithm to integrate the advantages of histogram-based and region-based methods. Since the problem of intensity based methods is that they don't consider any spatial information, we integrated these methods with a new region-based algorithm to take into account spatial information. Both intensity and neighborhood information were used in the segmentation process. The proposed algorithm consists of four steps after preprocessing steps: foreground/background thresholding, disconnecting the brain from non-brain tissues, region dividing, and image clustering. Firstly, the brain tissues were extracted from remained non-brain tissues in two steps. Then the extracted brain is devided into different sub-regions as homogeneous as possible using a new region deviding procedure. Finally, in the proposed image clustering step the brain image is classified into three tissue types. Fig 1, presents an overview of the first proposed method.

### 2.1 Preprocessing steps

Preprocessing step is a set of image preparation methods that improve the image for further processing steps.

#### 2.1.1 Brain extraction from MRI

In MRI analysis, defining brain from non-brain tissues is an important step. The problem is that the measured intensities of predominant tissue types (WM, GM and CSF) may overlap other tissue types, such as bone, skin, fat, muscle, which complicates the segmentation process. Therefore the brain surface skull stripping is one of the important preprocessing stages of brain MRI. In this study, we removed the skull and other extraneous tissues of brain applying the Brain Surface Extractor (BSE) that is publicly available at http://neuroimage.usc.edu/bse/.

#### 2.1.2 Noise reduction

Magnetic resonance images are corrupted by Rician distribution that arises from complex Gaussian noise in the original frequency domain measurements. Median filter is one of the most popular nonlinear spatial filters for noise reduction that is more efficient than convolution when the purpose is to preserve borders and decrease noise simultaneously. This method is simple, computationally efficient and has good denoising power.

The median filter replaces the value of a given pixel with the median pixel value within a region of interest (ROI). A median filter with properly selected window size can smooth the noise in the original image. It may also virtually eliminate the main tissues information from the MR image. Therefore, there will be a trade-off between noise reduction and the preservation of information from image. Clearly, by increasing the size of the median window, both noise signals and signals from main tissues are being suppressed [22]. The operation of median filtering technique is presented as:

$$\text{f}\left(\text{x},\text{y}\right)={\text{Median}}_{\left(\text{s},\text{t}\right)\in }\;S_{xy}\left\{\text{g}\left(\text{s},\text{t}\right)\right\}$$ (1)

where S_xy_ is the set of coordinates in a sub image window which is centered at (x,y), and the median is the value of the window respectively.

#### 2.1.3 Image normalization to compensate for the bias field effect

**Fig 2:**
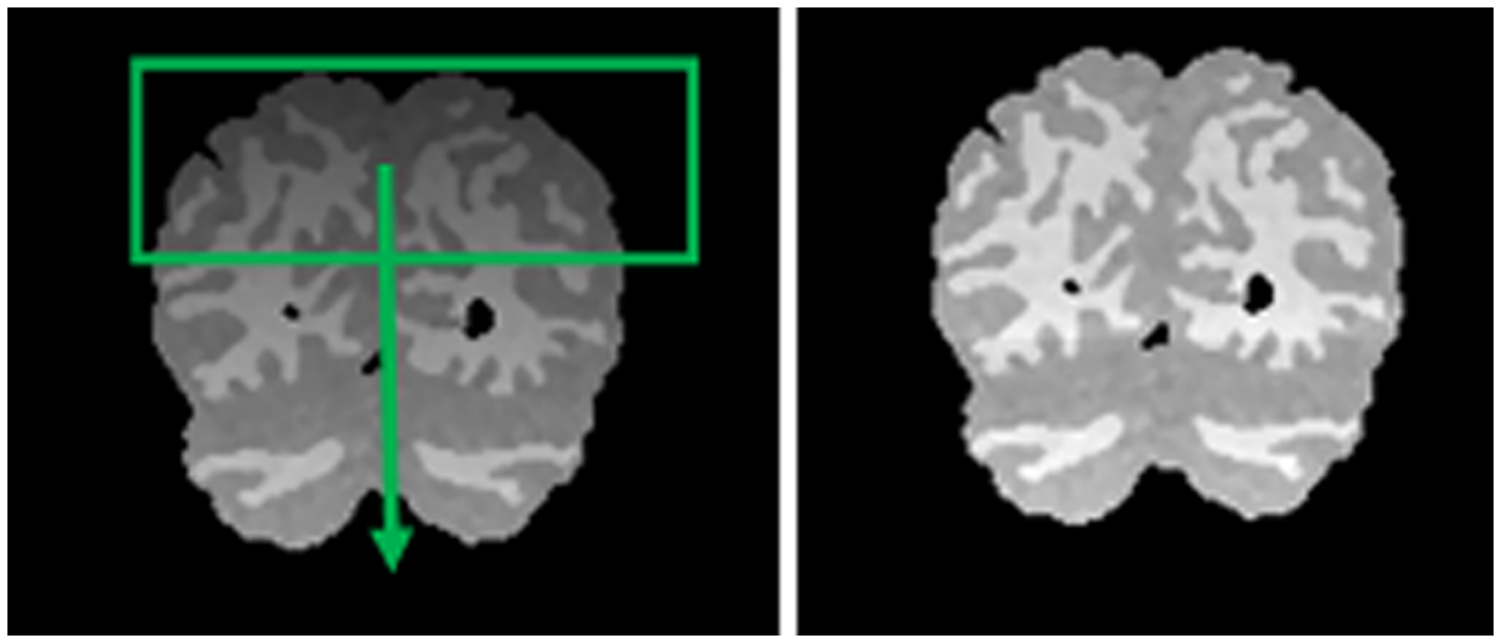
From left to right; Original image with bias field, Bias field corrected image. The green rectangle indicates the sliding window which is moving vertically from the top to the bottom of the image.

In order to segment MRI images using image-processing methods, undesirable signals and noise must be suppressed before segmentation. Since one of the major challenges is removing the bias field, a normalization step is required to remove low frequency magnetic field variations within the captured MR Images in order to regulate image brightness and contrast while preserving details. In this study a new method was proposed to reliably compensate bias field effects. A description of the steps of the proposed method is as follows:

In this method, the sliding window was applied to move vertically on each MR image to compensate the bias field effects using histogram analysis (See Fig 2). However, the size of the sliding window can vary during this process and covers lines of the image to calculate the normalization factor. But only the line in the middle of the window (mid-line or interested-line) was replaced by the new normalization factor. While the sliding window is moving from the top to the bottom of the image, a new normalization factor is calculated and applied to each interested-line of each slide sequence. Consequently, each mid-line was normalized based on the pixel data gathered from both previous and next consecutive lines.

At the start point of this process, the interested-line was considered the first line of the image at the top. Consequently, the sliding window needs to expand from the top while the process is in progress and the window is coming down to reach the bottom of the image.

In order to calculate the normalization factor, all the pixels within the region of the sliding window were considered as the input data. When the minimum and maximum pixel values of the mid-line were obtained, the mid-line pixel data was then stretched to its maximum data resolution (8-bit, 0~255) as presented below. Eq 3 indicates the locally normalization via a reusable sliding window as follows:

PN=((P−Pm)*RM)(PM−Pm) (2)

where *P*_*N*_ is the normalized pixel value, p is the pixel value, *P*_*m*_ is the minimum intensity value in the midline, *P*_*M*_ is the maximum intensity value in the midline and *R*_*M*_ is the maximum value of pixel bit depth (R = 8-bit is 255). Finally the image was locally normalized using the proposed method.

Before the local normalization step, to improve the brightness of the MR image, the standard deviation of all pixel values in the sliding window (SD_1_) was calculated in addition to the standard deviation of the mid-line (SD_2_). In the case where X takes random values (*x*_*1*_, *x*_*2*_,…, *x*_*N*_) and each value having the same probability, the standard deviation was defined as follows:

$$\text{SD}=\;\sqrt{\frac{1}{\text{N}}\sum_{\text{i}=1}^{\text{N}}{\left({\text{x}}_{\text{i}}-\;\mu \;\right)}^{2}}\;,\text{ where }\mu =\;\frac{1}{\text{N}}\;\sum_{\text{i}=1}^{\text{N}}{\text{x}}_{\text{i}}\;$$ (3)

All the pixels in the mid-line need to be shifted to the desired offset, where SD_2_ meets the SD_1_ value. At this point, the difference of SD_1_ and SD_2_ was added to the pixel values of the mid-line to compensate for the bias field effect.

### 2.2 Brain Segmentation using a Hybrid Method

In this paper a hybrid method which is a combination of histogram-based and region based methods was proposed to segment the MR brain images. The proposed method is a four-stage process which is explained as follows.

#### 2.2.1 Foreground/ background thresholding

**Fig 3:**
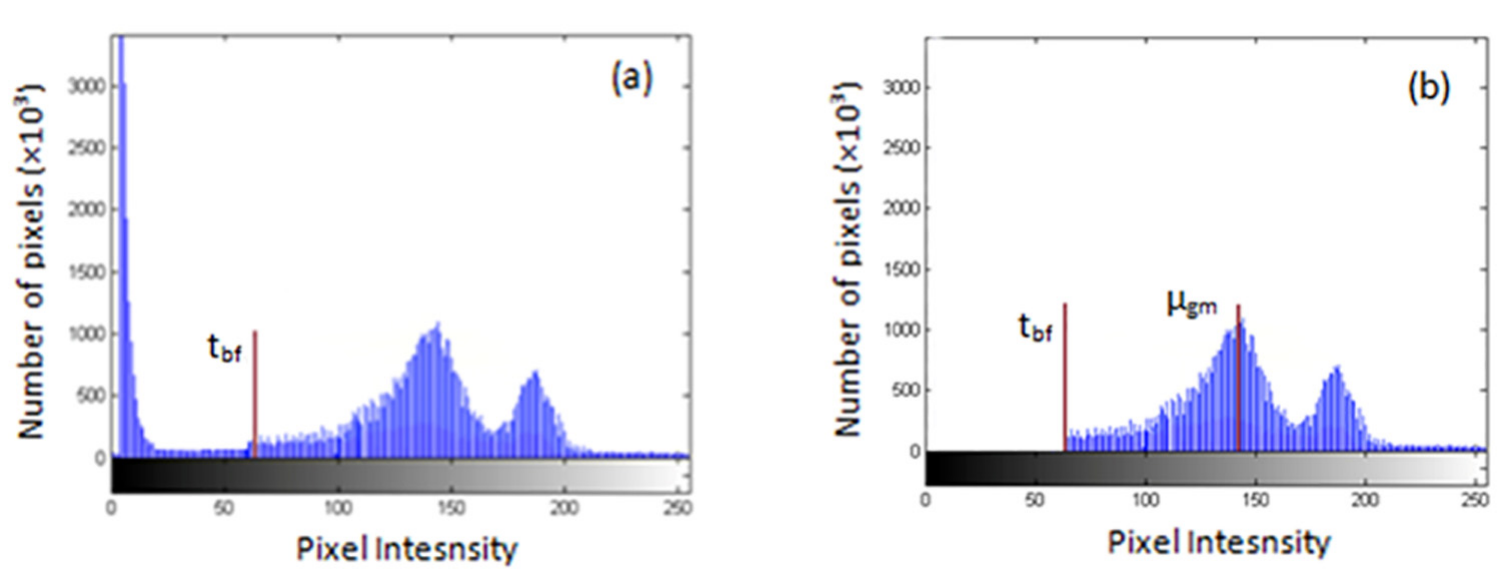
Histogram of the image. (a) Histogram of the real data. T_bf_ is a threshold that segments the background from the foreground. (b) Histogram of the same data as in (a) but after thresholding the background/foreground pixels.

After the skull stripping in the preprocessing step to remove the remained non-brain tissues, an automated method was proposed to remove the foreground /background. This stage is foreground /background thresholding to separate the background and foreground tissues. The threshold (t_bf_) was defined based on an analysis of the image histogram [23, 24]. As shown in Fig 3(a), the histogram of a real MRI image is presented. Any pixel with signal intensity lower than a defined threshold have been removed. These removed pixels included those contributed by very low-intensity components such as air. The histogram of resultant image (I_1_) is demonstrated in Fig 3(b), in which the low-intensity peak in Fig 3(a) has been deleted.

#### 2.2.2 Disconnecting the brain from non-brain tissues

The purpose of this stage is to disconnect the brain from non-brain tissues based on the following assumptions: The first one is that brain region is connected to the skull and neck tissues through rare connections in the MR image. Most of these connections indicate a lower intensity than GM intensity, but a small portion has a similar intensity. The second assumption is that the brain is the largest connected component of the head MR image [23]. The brain is extracted from the remained non-brain tissues by the following steps:

1. Three peaks in the image histogram were searched and located. Based on the assumption that each of the tissues has a Gaussian distribution the intensity value of the second peak was taken as a mean intensity value for the second peak or GM (μ_gm_).
2. The binary mask image (M_1_) was created from image I_1_ (resultant images following the application of Step 1), using a threshold (t_s_) at a level lower than μ_gm_. In the I_1_ image, the pixels with intensities smaller than t_s_ were set to 0, otherwise they were set to 1. Based on the result of repetitively testing different images, the threshold value was selected as:
  $$t_{s}=\;t_{bf}\;+\;\;\frac{2}{3}\;(\mu_{gm}\;-\;0.9\;t_{bf})$$ (4)
  where t_bf_ is the threshold, which segments the foreground from background.
3. A morphological binary opening was used to binary mask applying a spherical structuring element with a radius of three pixels. This stage disconnected the brain from the remained skull and other tissues.
4. In this stage a connected component processing was performed on the resultant images. The largest connected component (brain) is kept and the remaining components (non-brain tissues) were removed.
5. Three steps of dilation were performed from the last step applying a spherical structuring element with a radius of three pixels.
6. The extracted brain image (I_2_) was created by pixel-to-pixel multiplication of I1 image and the resultant mask image (M_1_).

$$I_{2}=I_{1}\;\cdot \;M_{1}$$ (5)

In image I_2_, the non-brain tissues were removed and the remaining image was dominated by the main tissues of the brain. In the next part the extracted brain is classified to different regions for further clustering step.

#### 2.2.3 Segmenting the image into several regions using an automatic region growing method

The basic function of region growing technique is to divide an image into non-overlapped regions. This study proposed an automatic region-growing algorithm based on the standard region growing technique that automatically selected some points as a seed region to continue the growth of the region according to certain rules. The process is recursive and in each stage, a pixel was joined to the specific region according to the uniformity criterion. This procedure was repeated until no more pixels are left to join the last region. Result of region growing algorithm should follow the constraints below:

- The total number of regions should form the brain image as a whole
- Each pixel should not belong to more than one region
- Each pixel in the brain should belong to a region

The region based segmentation method is explained below. In this section the brain image was segmented into different regions in four steps as follows:

1. Consider the brain as a big region and add it to a dividing list.
2. Region dividing step: Segment each brain into several sub-regions.
3. Repeat step until the dividing process is finished.
4. Collect all the sub-regions and assign each region to a global color.

One approach to extend the seed growing technique is to automate the selection of seed point. In the proposed method the first pixel with the intensity level greater than the predefined value (t_b_), is the first interested pixel for the seed growing process. Every pixel has been traversed in a zigzag way from top-left corner of the image to the bottom-right corner.

$$t_{b}=\;2/3\left|\mu_{gm-}t_{bf}\right|$$ (6)

**Fig 4:**
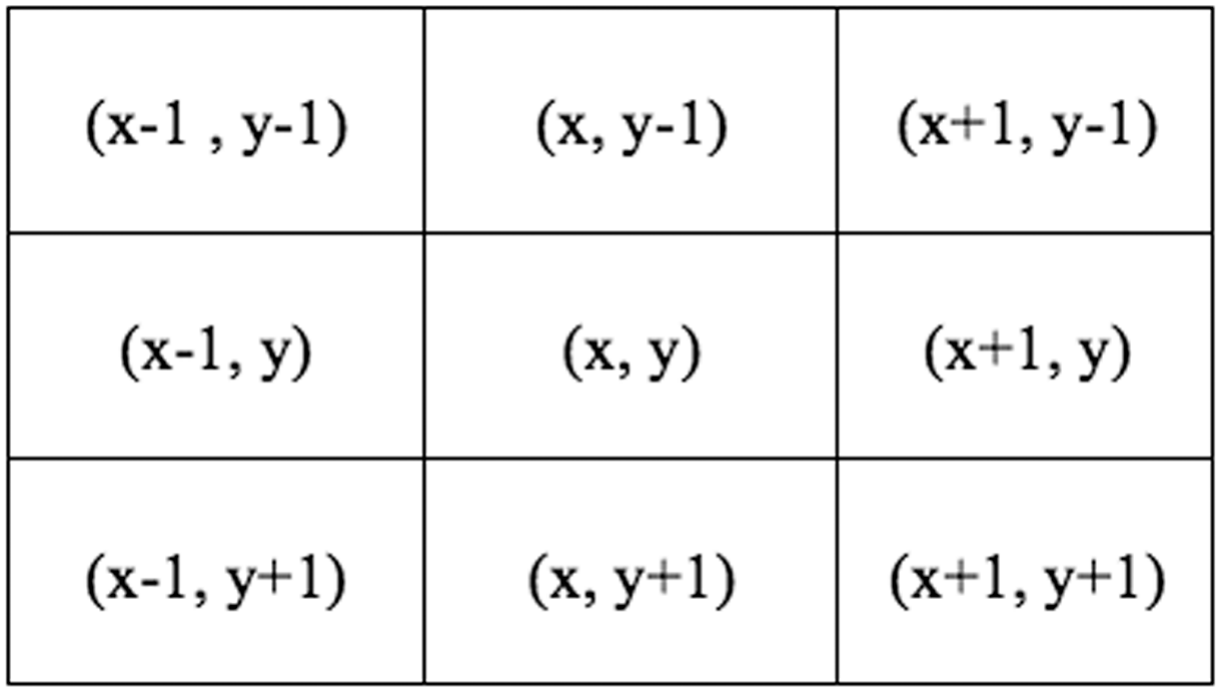
Illustration of 8-connected neighborhood or second order neighborhood of the interested pixel (x,y).

For each interested pixel (x,y), first the second-order neighbours (see Fig 4) were merged with each other by comparing their intensity by predefined value. Then the other neighbours joined the region. In this study, what is needed is not the definition of specific thresholds, but the determination of trends. In other words, in each iteration the evaluation process depends on the difference between the intensity level of interested pixel and its neighbors. Therefore, the value of homogeneity criterion changes at each step (see Eq 7).

$$if\;\{\begin{array}{c} \left|I_{i}-I_{n}\right|<c\;\;\;=>\;the\;\text{ neighboring}\;\text{pixel}\;\text{is}\;\text{added}\;\text{to}\;\text{the}\;\text{region}\\ \;\;\;\left|I_{i}-I_{n}\right|>c\;\;\;=>\;the\;\;\text{neighboring}\;\text{pixel}\;\text{is}\;\text{not}\;\text{added}\;\text{to}\;\text{the}\;\text{region} \end{array}$$ (7)

where I_i_ is the intensity level of interested pixel, I_n_ is the intensity values of neighboring pixel and c is the predefined value. This method circumvents the problem of using constant thresholds by replacing them with the local thresholds.

If the neighbors of seed points are in the range of defined value they will be joined to the generated region and replaced with the masked color. The method is based on non-masked color pixels as start points by traversing pixels in a zigzag way. Consequently, the next candidate seed points were selected under this condition, containing a pixel value other than the masked color.

In the next step the regions were labeled with the global color (*AV*_*1*_, *AV*_*2*_,…, *AV*_*m*_*)* that is the average intensity level of the pixels within that region. We considered the brain image as a big region. In each iteration, a sub-region was extracted and the processed area is replaced with the related 'AV'.

The segmentation process continues until there is no pixels left other than the masked color regions. Each extracted sub-region (R_1_, R_2_,…, and R_m_) was given a global color for further processing. The result of this stage is a brain image which is divided into different regions based on the uniformity criterion. The average intensity values of corresponding areas (RI) were computed, with $\left(X_{i}^{c},Y_{i}^{c}\right)$, the centroid of R_i_.

$$AV_{m}=\frac{\;\;\Sigma_{J=1}^{Nm}\;I{\left(X_{i}^{c},Y_{i}^{c}\right)}_{mj}}{Nm}$$ (8)

Let N and m be the number of pixels in the corresponding region and the number of regions respectively and $I{\left(X_{i}^{c},Y_{i}^{c}\right)}_{mj}$ demonstrates the intensity of each pixel in the *m*th region.

Since the difference of 'AV ' values between some neighbor regions may be negligible and colors look similar, these neighboring similar regions were merged together. In the next section, the color assigned regions were clustered into three main tissue classes.

#### 2.2.4 Clustering the sub-regions into three tissue classes

Since the segmented result contains very small regions in a diversity of intensity levels, we can easily cluster the regions into three groups. Based on the visual characteristics of white matter, which is the brightest part of brain image, it is considered to be segmented by the brightest gray regions. However, the gray matter is a little darker than the white matter but there is no sharp edge between these two parts of the brain that can easily be distinguished.

**Fig 5:**
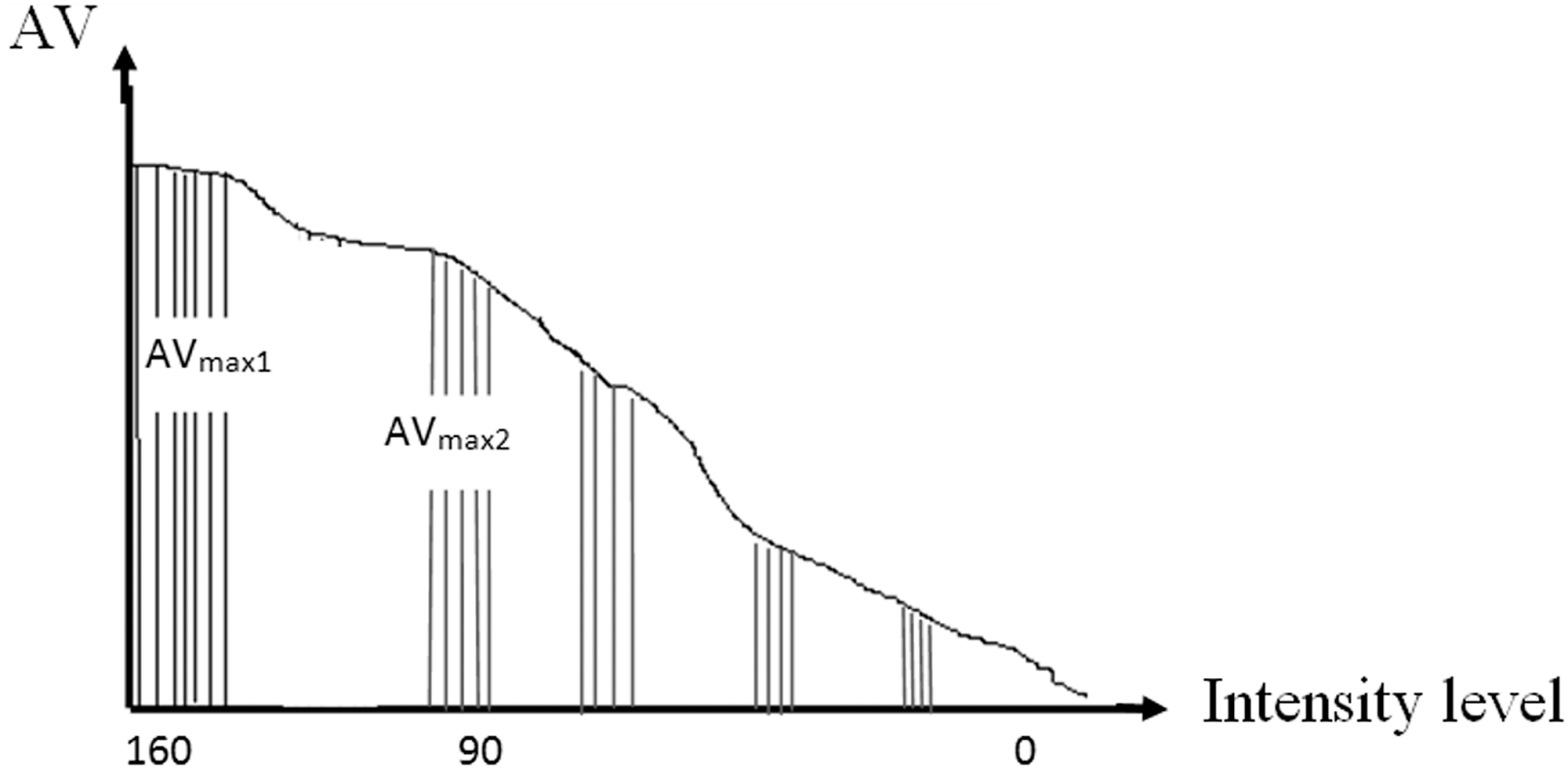
Finding two peaks of sorted histogram.

First, we generated a histogram of predefined 'AV ' values of sub-regions and then used quick sort algorithm to find the two peaks of the histogram (AV_max1_, AV_max2_) (See Fig 5).

**Fig 6:**
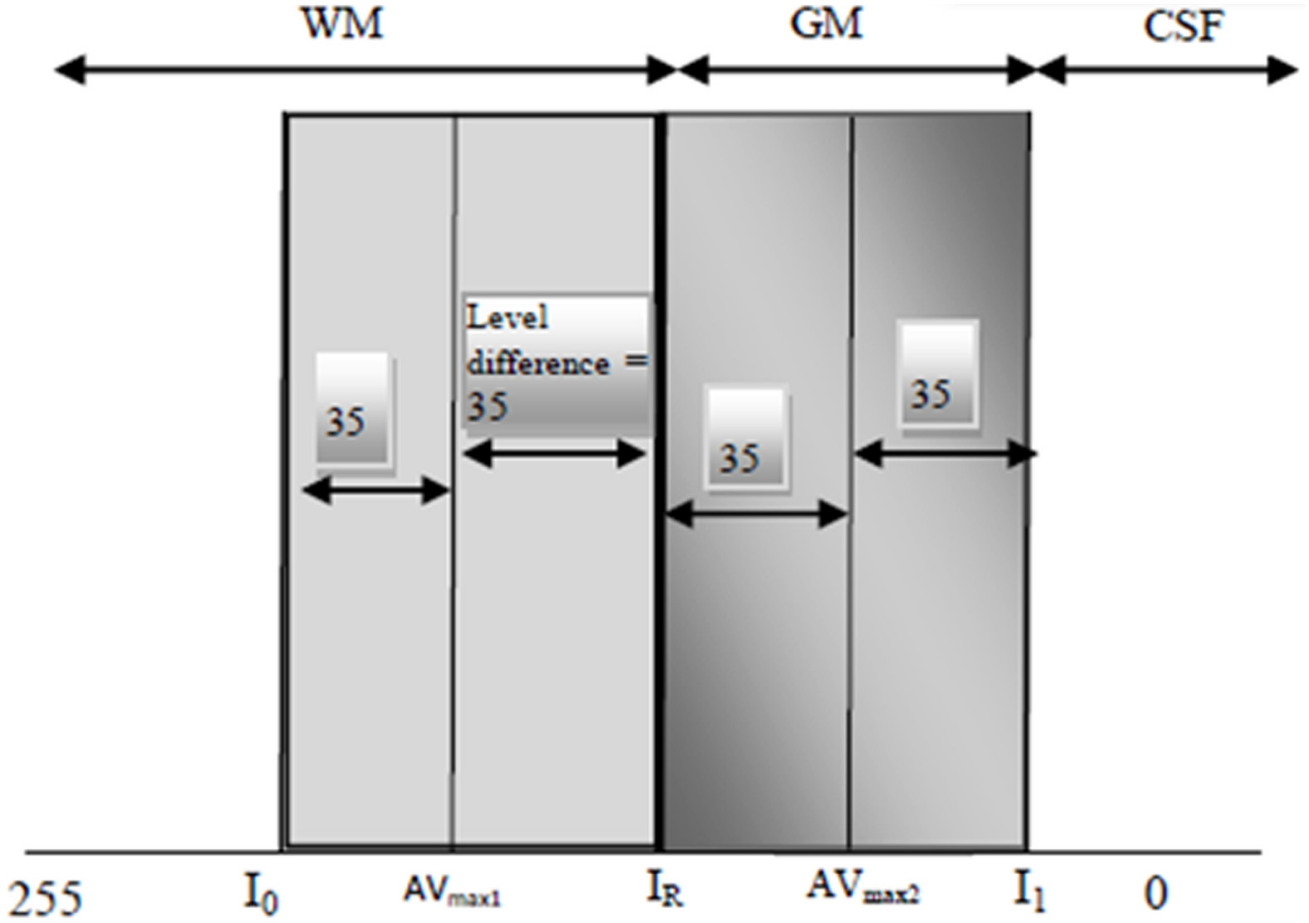
Illustration of clustering the sub-regions into three tissue classes.

By defining the first two peaks of histogram from the highest level (white = 255) to the lowest level (black = 0), the threshold values for clustering the sub-regions can be defined. A threshold value $\left({\text{I}}_{\text{R}}=\;\frac{AV_{max1-}AV_{max2}}{2}\right)$ was determined as a reference to distinguish GM and WM (See Fig 6). Consequently, every single image has different threshold values from another image. By using threshold values, the sub-regions can be easily clustered into: WM where the global intensity of every sub-region is equal or greater than I_R_ and GM where it is between the two thresholds (I_R_, I_1_). All other pixels that are not included in this range were considered as CSF.

## 3 Experimental Result and Discussion

Since the interest in the computer-aided, quantitative analysis of MR images is growing, the need for the evaluation of such methods is also increasing. Unfortunately, there exists no gold standard or ground truth for the analysis of results. The ground truth is human segmented images or anatomical models that provide main tissue class labels for each pixel. The Brainweb and IBSR websites provide a solution to the evaluation problem in the form of simulated and real databases [25]. Brainweb is a dataset with several versions of simulated images, at different noise levels and with different degrees of bias field. They are available at the website http://www.bic.mni.mcgill.ca/brainweb/ of the McConnell Brain Imaging Center at Montréal Neurological Institute (MNI) [26–28]. Moreover, a ground truth of the model is provided by the simulator in the Brainweb dataset. Simulated brain images are generated using an MRI simulator developed at the McConnell Brain Imaging Centre that allows users to achieve realistic brain MRI and independently control different acquisition parameters.

In this study, 18 simulated images were applied from Brainweb dataset with different level of noise and bias field. In this dataset each MR Image is provided with a ground truth. The proposed method was also applied to real images from IBSR dataset. IBSR is a publicly available dataset which provides real brain images for different acquisition modalities. The IBSR has supported a collection of T1-weighted real MRI volumes [25]. These image datasets and their expert segmentations are publicly available at (http://www.cma.mgh.harvard.edu/ibsr/).

**Fig 7:**
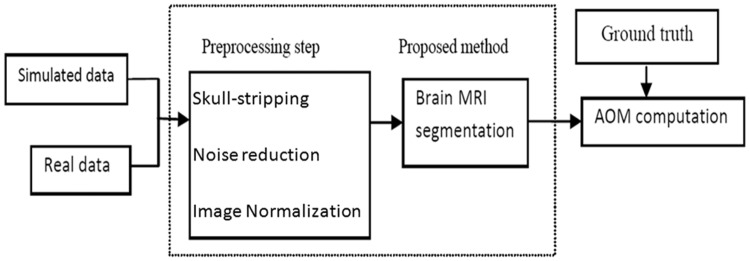
Overall assessment of the proposed method.

In order to investigate the performance of the proposed technique in terms of accuracy, AOM index was applied as a metric to assess the performance of segmentation method. AOM is calculated for each tissue of each volume compared to ground truth [29, 30]. The overall evaluation procedure is presented in Fig 7. The AOM is computed as follows:

$$\text{AOM}\left(\text{A },\text{B}\right)=\frac{2\left|\text{A}\cap \text{B}\right|}{\left(\left|\text{A}\right|+\left|\text{B}\right|\right)}$$ (9)

A displays the set of results achieved by the proposed method and B indicates the set of the ground truth dataset. These values reach a value of 1.0 for the results that are very similar and is near 0 when they share no similarly segmented pixels.

To verify the performance of the proposed method, two sets of experiments were performed on real and simulated data. These data sets were selected because they have various levels of artifacts and they have also been used in published studies. Since in these cases the ground truth (expert segmentation or anatomical model) is accessible, it is possible to have a quantitative evaluation of the performance of the technique.

### 3.1 Experimental Results and Evaluation with Simulated Data set

**Fig 8:**
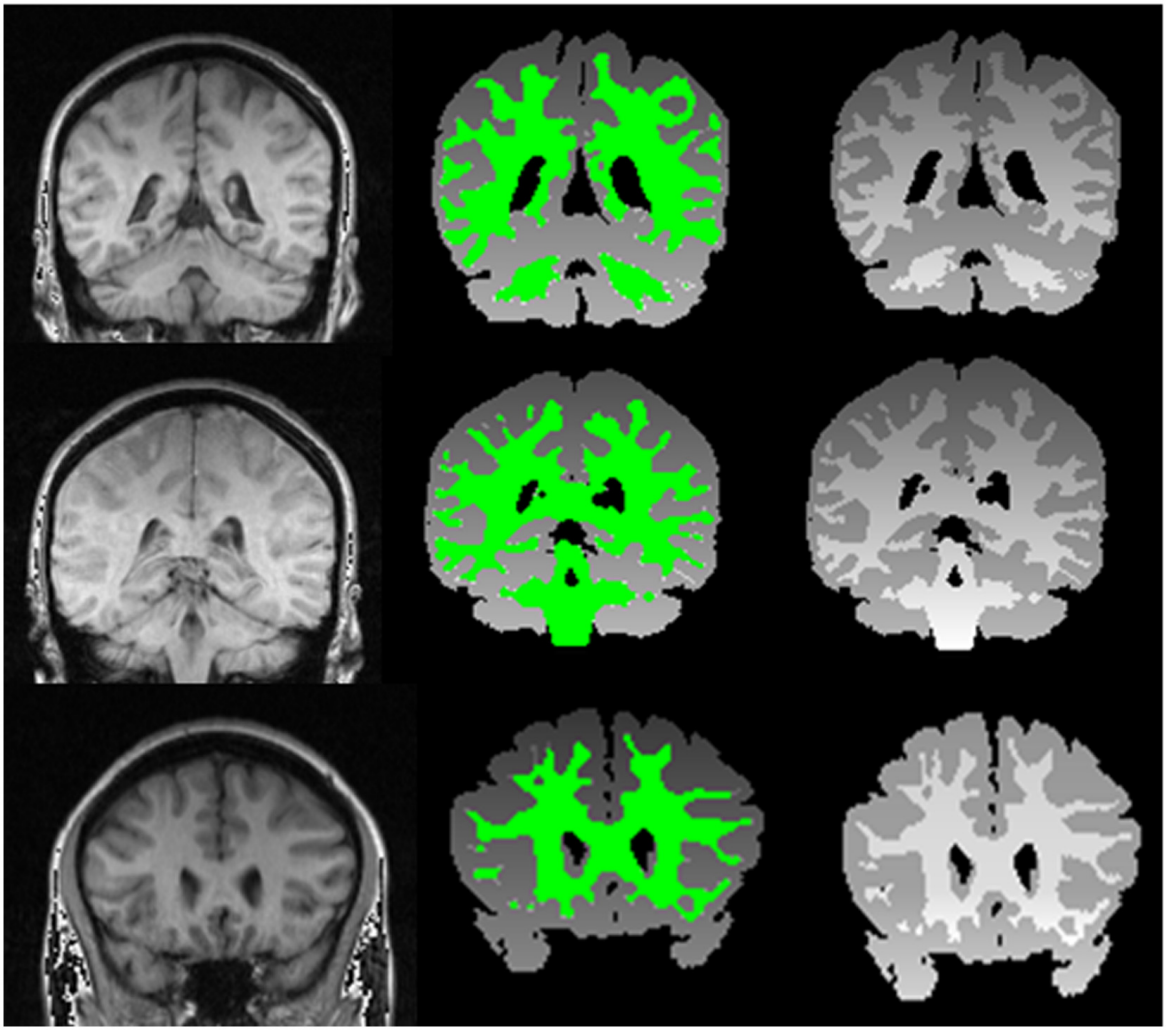
The first row from left to right indicates original images with 0% noise and 20% bias field, segmentation result and ground truth. The second row from left to right displays original images with 3% noise and 20% bias field, segmentation result and ground truth and the third row demonstrate original image with 9% noise and 40% bias field, segmentation result and ground truth.

For visual evaluation, different images were selected and the hybrid method was applied on them. The evaluation was started from the easiest case from Brainweb dataset with the following parameter settings: bias field: 20%, noise: 0% (See). Then in order to test the accuracy of the proposed method on the images with high level noise, MRI images with 3% noise and 20% bias field were used as our test samples (See Fig 8). Finally, the most difficult condition was tested with the following parameter settings: bias field: 40% and noise: 9% shows three types of images and their segmented results [31]. The first row presents images with 0% noise and 20% bias field, the second row displays images with 3% noise and 20% bias field and the third row demonstrates images with 9% noise and 40% bias field.

**Fig 9:**
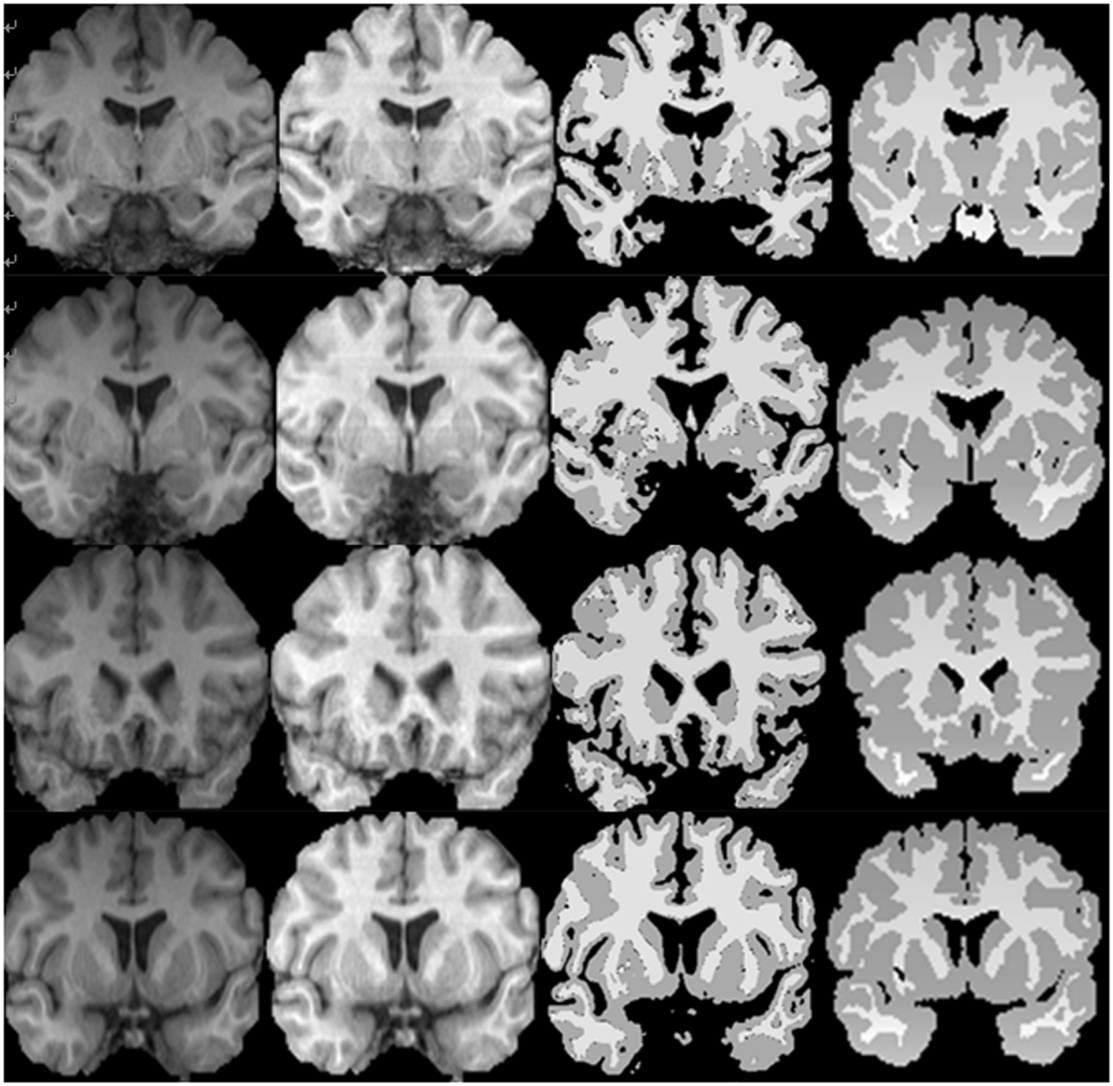
From left to right; The first column show original images from the simulated data, second column represents normalized images, the third column demonstrates the results of segmented WM and GM obtained by the proposed method (3% noise and 20% RF level), The last column indicates the ground truth.

Fig 9 consists of four slices (from slice #40 to slice #43) with 3% noise and 20% bias field levels, bias field corrected images, the results of the proposed method and their ground truth.

More quantitatively, the AOM similarity index for different levels of noise and bias field are given as follows. Since the ground truth includes only internal CSF while the proposed technique also defined sulcal CSF, the results for CSF were not reported. Table 1 demonstrates that the overal AOM measurements of WM and GM in different levels of noise and bias field are 0.93 and 0.92, respectively.

**Table 1:** Classification results on Brainweb T1-weighted MRI data in different levels of bias field and noise (pn).

MRI volume	WM	GM
Pn = 3%, rf = 0%	0.96	0.94
Pn = 9%, rf = 0%	0.95	0.93
Pn = 3%, rf = 40%	0.92	0.91
Pn = 9%, rf = 40%	0.92	0.92

To point out the contribution of the proposed framework, it would be compared with commonly used methods in the literature which are both region based and non-region based algorithms in different levels of Rician noise and inhomogeneity, as shown in Tables 2 and 3. The region based methods are Ayman et al. [32], Del-Fresnoet al. [30] and Yu et al. [14] methods and non-region based algorithms are the well established methods including FSL [33] an SPM [34, 35]. Tables show the AOM of WM and GM, respectively, after performing proposed algorithm to the test image. The AOM values of other methods in Tables 2 and 3 are based on free available reference software and published papers.

**Table 2:** The AOM for WM segmentation of the simulated databases with 3% and 9% noise and 0% and 40% bias field.

Noise	3%		9%	
RF	0%	40%	0%	40%
Ayman et al.	0.91	0.90	0.90	0.90
Del-Fresno et al.	0.94	0.89	0.91	0.87
Yu et al.	0.90	0.90	0.88	0.88
FSL	0.96	0.91	0.94	0.90
SPM 5	0.93	0.90	0.91	0.88
Proposed Method	0.96	0.92	0.95	0.92

**Table 3:** The AOM for GM segmentation of the simulated database with 3% and 9% noise and 0% and 40% bias field.

Noise	3%		9%	
RF	0%	40%	0%	40%
Ayman et al.	0.90	0.90	0.90	0.89
Del-Fresno et al.	0.90	0.89	0.90	0.86
Yu et al.	0.89	0.90	0.89	0.88
FSL	0.94	0.90	0.92	0.89
SPM 5	0.91	0.90	0.89	0.87
Proposed Method	0.94	0.91	0.93	0.92

Moreover, a comparison with other segmentation techniques is presented. Tables 2 and 3 show the AOM of WM and GM, respectively, after performing the proposed method to the T1-whighted MRI data.

The higher the AOM values, the more accurate the result. The results of proposed algorithm show satisfactory results in different levels of noise and bias field. With the AOM of WM and GM at noise levels of 3% and 9% and (RF = 0%, 40%) the proposed method has desired performance because the AOM values are 1% to 6% higher than other methods. Tables summarize values of the quality indexes computed in brain MR Images with different levels of artifacts. In particular, although the segmentation quality logically decreased in the presence of bias field and noise, the accuracy of the proposed method is satisfactory even compared with the other segmentation techniques. Furthermore the results present that, in spite of the anatomical complexity of the segmented object, the proposed technique can obtain high quality results. In WM segmentation the average AOM value of our algorithm is 0.93 that is superior to the other methods ranging from 0.89 to 0.92 except FSL method. In low level of bias field, FSL method is superior to our method while the proposed method is superior at high level bias field because of the proposed normalization method in section 2.1.3. The average AOM value of the proposed algorithm in GM segmentation is 0.92, which is substantially superior to other techniques ranging from 0.88 to 0.91. In general the results of WM segmentation are better than that of GM. The reason is explained in the next section. A more quantitative assessment of the results from the 18 real volumes is given in the following section.

### 3.2 Experimental Results and Evaluation with Real Data set

**Fig 10:**
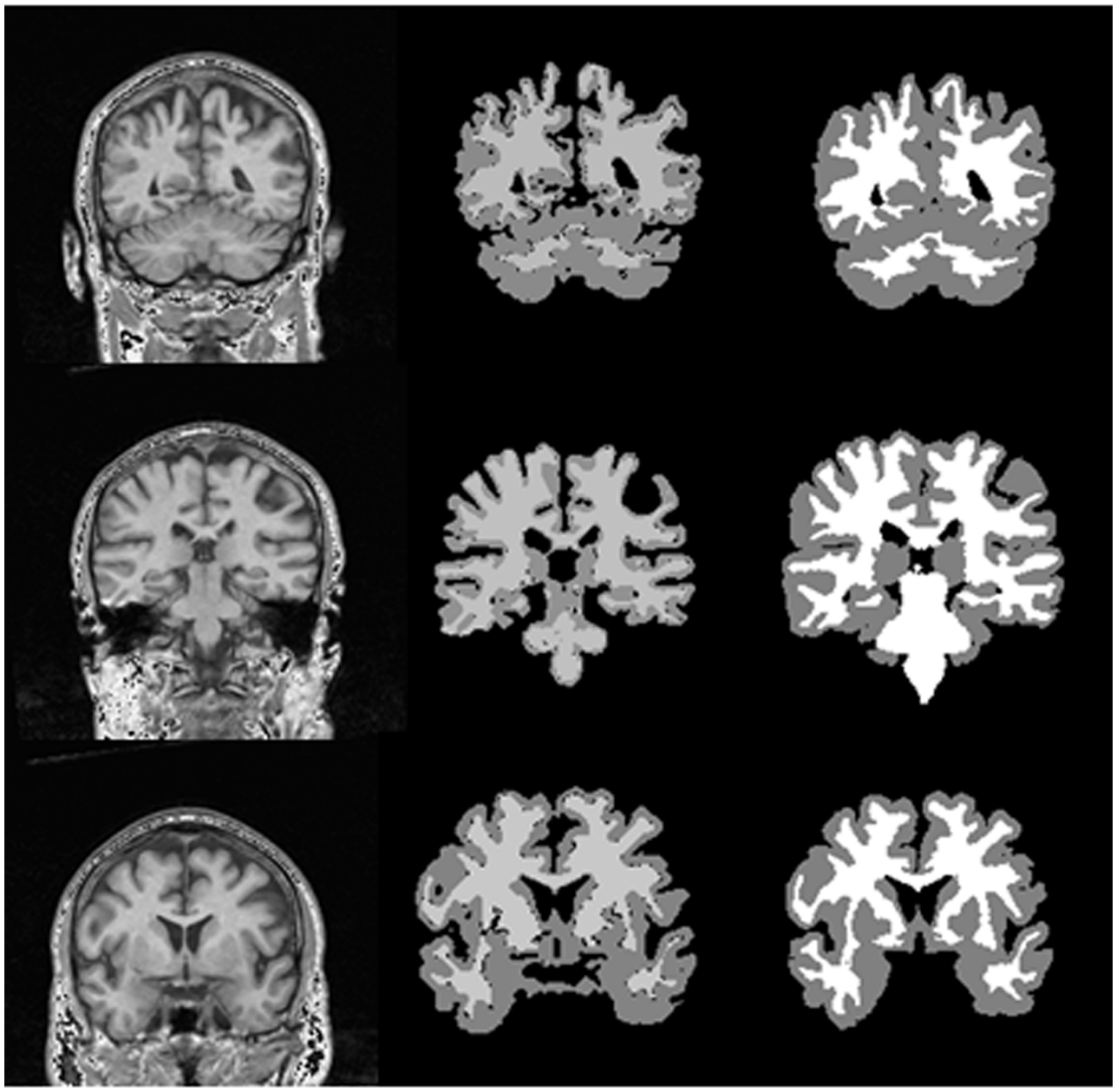
From left to right; first column presents the brain image from the IBSR dataset, second column displays segmentation results of the proposed method and third column presents the expert segmented images.

**Fig 11:**
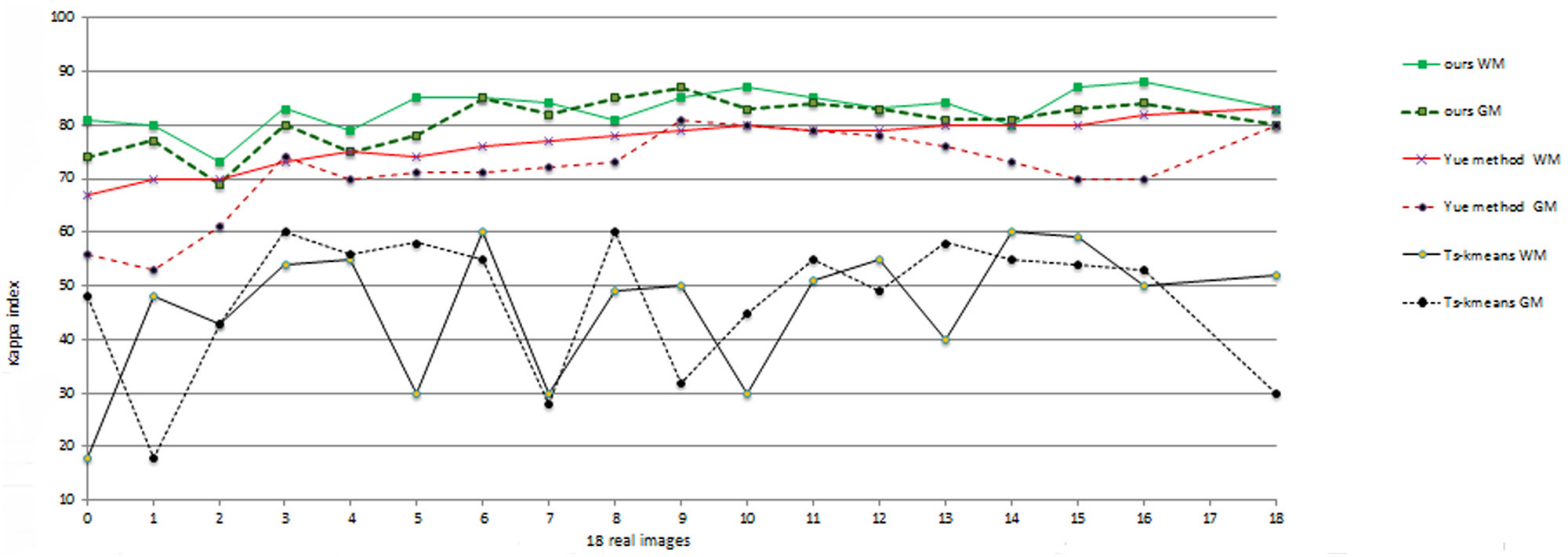
Comparison of WM and GM segmentation results using proposed technique, Yue method and Ts-kmeans. The vertical axis displays the AOM and the horizontal one represents the volume number.

The proposed method was also applied to real images from IBSR dataset. The IBSR has supported a collection of 18 T1-weighted real MRI volumes with their expert segmented scans. In Fig 10, the results of the proposed method on three normal volumes from IBSR are demonstrated. In addition, the images have also been used by other researchers [14]. The results of segmentation applying six techniques reported in the literature can be directly achieved from IBSR website [14]. This has allowed us to more easily investigate the performance of the proposed method in comparison with existing algorithms. In Fig 11, the comparison of our results on the 18 normal brain images using the proposed framework is presented including Yue method (A hybrid region-boundary model) [14] and Ts-kmeans technique that is one of the six techniques mentioned in [14, 36]. The other five techniques present almost the same performances as the Ts-kmeans [14].

The horizontal axis displays the volume number and the vertical axis indicates AOM values. We observed from Fig 11 that:

1. The overall AOM value for white matter segmentation using our technique is 0.82 which is substantially superior to 0.78 of the Yue method and those of the six other methods ranging from 0.48 to 0.56. For gray matter segmentation, the overall AOM of the proposed method is 0.80, also higher than the other methods, the AOM of which is between 0.55 and 0.71.
2. The proposed method is more robust in comparison with other techniques. Taking white matter classification as an example, the AOM values of our technique range from 0.74 to 0.87, with a difference of 0.13 between the minimum and maximum values. Comparatively, the difference between the minimum and maximum AOM of other techniques is about 0.6, implying that the performance of these algorithms is rather dependent on the type of images [14].
3. The results of white matter segmentation are better than that of gray matter. The first reason is that gray matter is much more convoluted than white matter. The other reason is that gray matter has a smaller region than white matter, and misclassification of only one pixel has a greater effect on gray matter segmentation than white matter.

## 4 Conclusion

In this study a hybrid brain MRI segmentation method was proposed to integrate the advantages of histogram-based and region-based methods. To the best of our knowledge, in the previous studies in the subject area, no such hybrid method had been developed for brain MRI segmentation. In the proposed technique, both spatial and intensity information were applied in the segmentation process. The proposed algorithm consists of four major procedures after preprocessing steps: foreground/background thresholding, disconnecting the brain from non-brain tissues, region dividing, and image clustering. In the first and second steps, the remained non-brain pixels are removed, and in the region dividing procedure, the image is divided into different sub-regions. In other words, given the set of initial seeds (S1, S2, S3,…, Sm), each step of region growing incorporates one additional pixel into one of the seed sets. The procedure is recursively repeated until all the brain pixels are assigned to the corresponding regions. The brain image is partitioned into different regions or areas as homogeneous as possible based on the given constraints. Finally, in the image clustering step the brain image is classified into three tissue types.

SRG is an easy-to-use, rapid, and accurate image segmentation method requiring neither training sets nor tuning parameters, including avoiding the complex post-procedures required by the latter method. It is also applicable to a wide range of image types. One of the drawbacks of the standard SRG procedure is that it is not a self-contained process, as it also requires the input of a few control points in the image known as seeds. These can be manually entered or can be the output of other techniques. Since in the proposed technique the task of choosing seed points has been automated, the method does not need user interaction to choose the first seed point. Another characteristic of the proposed approach is that MR images have been normalized before the segmentation process using a proposed local normalization method.

The method has been tested on the complex anatomical structures from simulated images, and real MRI volumes. Quantitative evaluation of the simulated images for the WM segmentation show superior performances. The comparison with existing techniques has demonstrated that the performance of the proposed approach can achieve higher K similarity index.

Further improvement in accuracy of GM segmentation is an interesting aspect to be investigated in future study. The authors hope that the proposed method can introduce new alternatives for the improvement of useful applications of medical segmentation methods.
